# Age and Gender Disparities in the Association of Long-Term Dietary Choline and Choline Compound Intakes with Incident Cognitive Decline in Middle-Aged and Older Chinese Adults: A Prospective Cohort Study

**DOI:** 10.3390/nu16234121

**Published:** 2024-11-28

**Authors:** Xiaofang Jia, Chang Su, Jiguo Zhang, Feifei Huang, Jing Bai, Fangxu Guan, Yanli Wei, Li Li, Yibing Liu, Jingang Ji, Wenwen Du, Yifei Ouyang, Xiaofan Zhang, Bing Zhang, Huijun Wang

**Affiliations:** 1 National Institute for Nutrition and Health, Chinese Center for Disease Control and Prevention, Beijing 100050, China; jiaxf@ninh.chinacdc.cn (X.J.); suchang@ninh.chinacdc.cn (C.S.); zhangjg@ninh.chinacdc.cn (J.Z.); huangff@ninh.chinacdc.cn (F.H.); baijing@ninh.chinacdc.cn (J.B.); guanfx@ninh.chinacdc.cn (F.G.); weiyl@ninh.chinacdc.cn (Y.W.); lili@ninh.chinacdc.cn (L.L.); liuyibing1206@163.com (Y.L.); 13730038263@163.com (J.J.); duww@ninh.chinacdc.cn (W.D.); ouyyf@ninh.chinacdc.cn (Y.O.); zhangxf@ninh.chinacdc.cn (X.Z.); zhangbing@chinacdc.cn (B.Z.); 2 NHC Key Laboratory of Public Nutrition and Health, Beijing 100050, China

**Keywords:** choline, phosphatidylcholine, glycerophosphocholine, cognitive decline, cohort study

## Abstract

**Background/Objectives**: The neuroprotective role of dietary choline during adulthood has not yet been conclusively proven. This study aims to investigate the influence of long-term choline and its constituent intakes on cognitive decline in the Chinese population. **Methods**: A total of 4502 subjects (≥55 years) with at least two waves of completed data and without cognitive decline at baseline were selected from the China Health and Nutrition Survey 1997–2018. Three consecutive 24 h dietary recalls were performed to collect dietary intake information for choline, phosphatidylcholine (PC), and glycerophosphocholine (GPC) measures. Several items from the Telephone Interview for Cognitive Status (Modified) were employed to perform a cognitive assessment. Cox frailty models were used to estimate hazard ratios (HRs) and 95% CIs. **Results**: A total of 783 participants developed cognitive decline during 26,080 person-years of follow-up. Cumulative average intakes of choline, PC, and GPC were 188.0, 126.7, and 17.1 mg/d, respectively. In the total population, after full adjustment, subjects in the lower (Q2), medium (Q3), higher (Q4), and highest (Q5) quintiles of dietary choline showed 27.8% (95% CI: 0.584, 0.894), 33.9% (95% CI: 0.522, 0.836), 23.0% (95% CI: 0.599, 0.990), and 29.3% (95% CI: 0.526, 0.949) decreases in the risk of cognitive decline compared to the lowest (Q1), respectively. Similar results were observed in PC but not GPC measures. Both higher choline and PC intakes induced a lower risk of cognitive decline for subjects ≥ 65 years at baseline (Q3 and Q4) and females (Q2–Q5). A marginally significant association of GPC was found for subjects ≥ 65 years (Q5) and males (Q4). **Conclusions**: These findings identify age and gender disparities relating to the protective associations of dietary choline, PC, and GPC with incident cognitive decline in middle-aged and older Chinese populations.

## 1. Introduction

Neurodegenerative diseases are among the various aging-related diseases and are becoming more prevalent as lifespans increase across the world [1]. Alzheimer’s disease (AD), a type of dementia, is a syndrome characterized by deterioration in cognitive functions beyond normal cognitive aging and produces negative influences in psychological, social, and physiological functions in addition to presenting a considerable economic burden in patients, family, and society [2]. Mild cognitive impairment (MCI) represents a preclinical, transitional stage between healthy aging and dementia, and it globally affects over 15% of community dwellers aged 50 years and older [2,3]. Due to there being no cure for dementia treatment, MCI has been consistently regarded as a “window” in which it is possible to intervene and delay progression to dementia [3]. 

As a transitional and reversible stage of early cognitive impairment, much attention has been focused on the identification of determinants of MCI or cognitive reserve [4,5,6]. The main modifiable risk factors for cognitive decline include socioeconomic, environmental and lifestyle factors as well as vascular-related risks [6,7,8]. Among them, nutrition is important for optimizing cognition and reducing the risk of MCI and AD [9]. The relationship between diet and cognitive function has been intensively explored with accumulating evidence supporting its role in the development of cognitive impairment from a food, nutrient, and dietary pattern perspective [10]. Choline, an essential nutrient for humans, is required for the synthesis of the neurotransmitter acetylcholine, the methyl group donor betaine, and phospholipids, and therefore it is involved in a broad range of crucial physiological functions across all stages of life [11]. In addition to de novo synthesis, the body’s choline requirements mainly obtained from the diet, in which choline is present in both water-soluble [free choline, phosphocholine, and glycerophosphocholine (GPC)] and lipid-soluble [phosphatidylcholine (PC) and sphingomyelin] forms [11]. Studies in rodent models showed that a high choline intake during gestation and early postnatal development improved cognitive function in adulthood, prevented age-related memory decline, and protected the brain from the neuropathological changes associated with AD [12,13,14]. In humans, whether dietary choline intake in adults also influences cognitive function and is associated with better cognitive performance and resistance to cognitive decline remains unclear.

Community-based longitudinal studies on choline intake and human cognition are scarce with mixed findings reported. A prospective population-based study using a sample of dementia-free men aged 40–60 years from the Kuopio Ischaemic Heart Disease Risk Factor Study reported those in the highest PC intake quartiles (>222 mg/d) at baseline compared with the lowest (<144 mg/d) had a 28% (95% CI: 1%, 48%) lower risk of incident dementia, while no association was observed for total choline intake. However, both choline and PC intakes were associated with a higher cognitive test performance level in verbal fluency and memory functions [15]. Another prospective study using data from adults free of dementia and stroke (mean age: 54.5 years) based on the Framingham Heart Study Offspring Cohort identified nonlinear relationships between dietary choline intake and incident dementia and AD, and it reported that a low choline intake was significantly associated with increased risks of incident dementia and AD compared with a moderate intake (≤219 mg/d vs. 220–516 mg/d for dementia; ≤215 mg/d vs. 216–552 mg/d for AD), but no effects were detected in the high-intake group (≥517 mg/d and ≥553 mg/d, respectively) [16]. Conversely, it was reported that neither dietary choline intake (OR = 0.94, 95% CI: 0.75, 1.17) nor total choline intake including dietary supplements (OR = 0.87, 95% CI: 0.70, 1.09) was associated with changes in cognitive test scores using data from older adults (≥60 years) from the National Health and Nutrition Examination Survey (NHANES) 2011–2012 and 2013–2014 waves [17]. Several small-scale randomized trials showed that choline supplements improved cognitive performance in older adults [18]. However, a systematic review involving 13 studies on the association of choline intake with neurological outcomes in adults did not find choline supplements improved cognition in healthy adults [19]. 

Taken together, the evidence of a potential neuroprotective effect of dietary choline in middle-aged and older adults was from community-based studies conducted in Europe and the USA, and findings were controversial, whereas studies in Chinese populations were lacking. Therefore, this study aims to fill the gaps by exploring the influence of dietary choline and its different forms on incident cognitive decline in middle-aged and older adults using longitudinal data from the China Health and Nutrition Survey (CHNS).

## 2. Materials and Methods

### 2.1. Study Population

The data for this study were derived from the CHNS, which was an ongoing longitudinal study established in 1989 through a collaboration between the Chinese Center for Disease Control and Prevention and the University of North Carolina at Chapel Hill. Detailed study protocols and profiles have been provided in previous publications [20,21].

We used data from the 1997, 2000, 2004, 2006, 2015 and 2018 waves, as cognitive measurements were consistently administered to adults aged 55 years and older in these years. Eligible participants were those with at least two waves of completed data on demographics, socioeconomics, lifestyle factors, disease history, anthropometric measurements, dietary assessments, and cognitive evaluations. We excluded individuals who exhibited cognitive decline at baseline (as determined by the first cognitive test), those with implausible energy intake [defined as >2.5 times the estimated energy requirement (EER) or <1/4 EER], and those with extreme outliers [mean ± 4 standard deviations (SD)] in the annual change in BMI [22]. In total, 4502 participants were included as the baseline sample and were followed for incident cognitive decline with a mean follow-up duration of 5.8 years.

### 2.2. Dietary Recalls and Cumulative Average Choline Intake

Dietary intake data were collected using three consecutive 24 h dietary recalls, which were conducted by trained investigators through face-to-face interviews. Participants provided detailed information about their food consumption, including the type and amount of food, cooking methods, and eating locations. In addition, daily household food inventories were used to track the consumption of household edible oils and condiments.

Food items were coded and analyzed to calculate the daily intake of energy and various nutrients, using the Chinese Food Composition Tables (2009, 2018 and 2019) [23,24,25]. Choline intake, including the specific choline compounds PC and GPC, was assessed using data from the 2008 USDA Database for the Choline Content of Common Foods [26], the 2018 USDA National Nutrient Database for Standard Reference, and values from Zeisel et al. [27]. To estimate the long-term habitual intake and minimize within-individual variation, we calculated the cumulative average intake for each participant up to each survey year prior to censoring or cognitive decline [28]. For example, the cumulative average choline intake in 2000 was based on data from 1991, 1993, 1997 and 2000. This cumulative average approach was also applied to assess PC, GPC and energy intake.

### 2.3. Cognitive Assessment

Cognitive performance was assessed using screening items from the Telephone Interview for Cognitive Status-modified [29], which was administered to participants aged 55 years and older in the 1997, 2000, 2004, 2006, 2015 and 2018 survey years. The cognitive tests included immediate and delayed recall of a 10-word list (10 points each), counting backward (2 points) and serial 7’s subtraction (5 points). The total score across all items ranged from 0 to 27 with higher scores indicating better cognitive function. Cognitive decline was defined as a score in the quintile of the cognitive test distribution with a cutoff of <9 points in this study [30].

### 2.4. Covariates

We collected information on potential confounders, including age, gender, education, household income, lifestyle factors, geographic location, community surroundings, and disease history. Age was divided into 55–64 years and ≥65 years. Education was grouped as ≤primary school, middle school, and ≥high school. Household income was categorized into year-specific tertiles (low, medium, high), as was the urbanization index [31] and total physical activity. Geographic location was classified into central, eastern, and western regions. Smoking and alcohol intake were dichotomized into yes/no categories. Participants with a self-reported history of hypertension, diabetes, myocardial infarction, or stroke were classified as having cardiovascular disease (CVD). BMI was calculated as weight in kilograms divided by height in meters squared with height and weight measured according to standard protocols [32].

### 2.5. Statistical Analysis

Descriptive statistics are presented as mean ± SD for continuous variables and as frequency (n, %) for categorical variables. Differences in baseline characteristics across quintiles of cumulative mean choline intake were analyzed using the Kruskal–Wallis test for continuous variables (due to skewed distributions), the Cochran–Armitage trend test for binary variables, and the chi-square test for categorical variables. If the Kruskal–Wallis test indicated significant differences, post hoc comparisons were made using the Student–Newman–Keuls method.

Cox frailty models with random intercepts for community [33] were used to estimate the association between higher quintiles of dietary choline intake and time to cognitive decline onset relative to the lowest quintile. Hazard ratios (HRs) and 95% confidence interval (CIs) were reported to account for clustering within communities. Follow-up time was calculated as the interval from baseline to either the first occurrence of cognitive decline or censoring (including loss to follow-up or the last visit where no cognitive decline was observed). 

Model 1 was adjusted for age, gender, education, household income, geographic location and urbanization level. Model 2 was additionally adjusted for alcohol intake, total physical activity and cumulative average energy intake. Model 3 was further adjusted for CVD history, BMI and baseline global cognitive score. Time-dependent covariates were used for cumulative average choline/PC/GPC/energy intake, age, household income, urbanization index, physical activity, alcohol intake, CVD status, and BMI in each model. 

Trend analyses across quintiles were performed by assigning participants the median value of each quintile and assessing the continuous association. Similar analyses were conducted for dietary PC and GPC intake adjusting for choline intake in Model 3. Given marginally significant interactions of age (*p* = 0.0560) and gender (*p* = 0.1106) with higher dietary choline intake in Model 3, and the well-established age and gender disparity in cognitive impairment [2,6], stratified analyses by age group (55–64 years vs. ≥65 years) and gender (male vs. female) were also conducted.

All statistical analyses were performed using SAS 9.4 (SAS Institute, Cary, NC, USA), and visualizations were created using R 4.3.2. A two-sided *p* value < 0.05 was considered statistically significant.

## 3. Results

### 3.1. Baseline Characteristics of Study Population

At baseline, the average age of 4502 participants was 62.3 years, and 51.5% was male (Table 1). A larger proportion of participants had lower educational attainment (49.1%) and resided in eastern China (45.1%). Additionally, 28.7% were current smokers, 31.6% consumed alcohol and 24.2% had a history of CVD. The average daily intakes of choline, PC and GPC were 188.0 mg, 126.7 mg and 17.1 mg, respectively. 

As dietary choline intake increased, age and BMI also tended to rise. Participants with higher choline intake had significantly higher energy, PC and GPC intakes as well as higher global cognitive scores across quintiles (all *p* < 0.05). The proportion of participants with lower educational attainment decreased, while those with higher education levels increased with higher choline intake. Similarly, there were significant changes in the proportions of participants across the low, medium and high categories of household income and urbanization across choline intake quintiles. Conversely, individuals with high levels of physical activity were less represented in the higher quintiles of choline intake, while the proportions of those with low or moderate physical activity levels increased. Participants from eastern regions of China were more likely to be in the higher choline intake quintiles. Furthermore, the proportions of males, alcohol consumers, and those with a history of CVD increased with higher choline intake.

### 3.2. Association of Dietary Choline and Its Compounds with Incident Cognitive Decline in the Total Population

**Figure 1:**
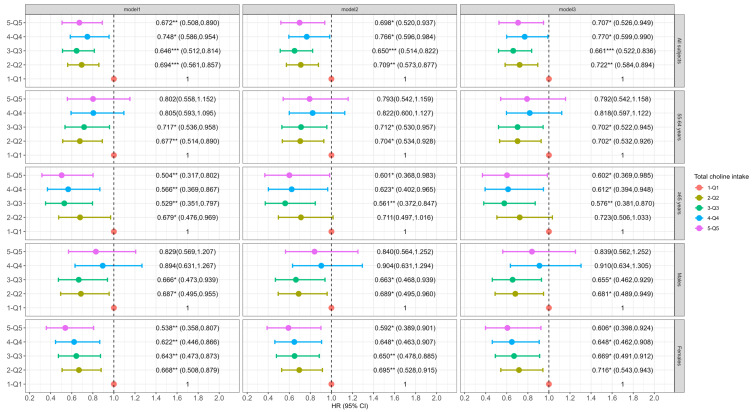
Association of dietary choline with incident cognitive decline in total subjects and grouped by baseline age and gender. The Cox frailty model was employed and results are shown as HR (95% CI). Model 1 adjusted for age, gender, education, household income, residence region and urbanization level. Model 2 additionally adjusted for alcohol intake, total physical activity and energy intake. Model 3 additionally adjusted CVD history, BMI, baseline global cognitive score. For gender-stratified analysis, gender was excluded in Model 1. * *p* < 0.05, ** *p* < 0.01, *** *p* < 0.001.

**Figure 2:**
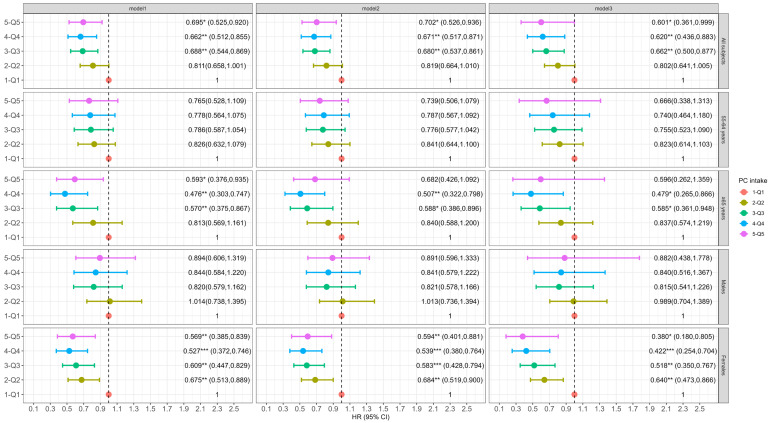
Association of dietary PC with incident cognitive decline in total subjects and grouped by baseline age and gender. The Cox frailty model was employed, and results are shown as HR (95% CI). Model 1 adjusted for age, gender, education, household income, residence region and urbanization level. Model 2 additionally adjusted for alcohol intake, total physical activity, energy intake and choline intake. Model 3 additionally adjusted CVD history, BMI, baseline global cognitive score. For gender-stratified analysis, gender was excluded in Model 1. * *p* < 0.05, ** *p* < 0.01, *** *p* < 0.001.

**Figure 3:**
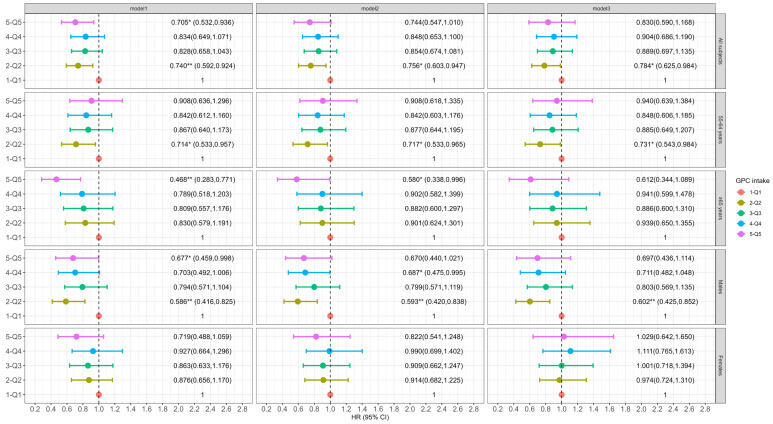
Association of dietary GPC with incident cognitive decline in total subjects and grouped by baseline age and gender. The Cox frailty model was employed, and results are shown as HR (95% CI). Model 1 adjusted for age, gender, education, household income, residence region and urbanization level. Model 2 additionally adjusted for alcohol intake, total physical activity, energy intake and choline intake. Model 3 additionally adjusted CVD history, BMI, and baseline global cognitive score. For gender-stratified analysis, gender was excluded in Model 1. * *p* < 0.05, ** *p* < 0.01.

Participants with a higher intake of dietary choline, PC or GPC (across quintiles) had a lower incidence of cognitive decline. Specifically, in the fully adjusted models, individuals in the second (Q2), third (Q3), fourth (Q4) and fifth (Q5) quintiles of dietary choline intake showed a 27.8% (95% CI: 0.584, 0.894), 33.9% (95% CI: 0.522, 0.836), 23.0% (95% CI: 0.599, 0.990), and 29.3% (95% CI: 0.526, 0.949) reduction in the risk of cognitive decline compared to those in the lowest quintile (Q1) (*p*-trend = 0.2563, Figure 1). Higher PC intake was consistently associated with a lower risk of cognitive decline in Q3 (HR: 0.662, 95% CI: 0.500, 0.877), Q4 (HR: 0.620, 95% CI: 0.436, 0.883), and Q5 (HR: 0.601, 95% CI: 0.361, 0.999) compared to Q1 (*p*-trend = 0.8465, Figure 2). For dietary GPC (Figure 3), only participants in Q2 had a significantly lower likelihood of cognitive decline compared to those in Q1 (HR: 0.784, 95% CI: 0.625, 0.984, *p*-trend = 0.4917).

### 3.3. Association of Dietary Choline and Its Compounds with Incident Cognitive Decline by Age at Baseline

Among participants aged 55–64 years at baseline, dietary choline intake in Q2 and Q3 was associated with a 29.8% (95% CI: 0.532, 0.926) and 29.8% (95% CI: 0.522, 0.945) lower risk of cognitive decline compared to Q1 (*p*-trend = 0.3606, Figure 1). No significant association was observed between PC intake and cognitive decline across quintiles after full adjustment (*p*-trend = 0.3399, Figure 2). However, for GPC, the participants in Q2 had a significant lower risk of cognitive decline relative to Q1 (HR: 0.731, 95% CI: 0.543, 0.984, *p*-trend = 0.1749, Figure 3).

For participants aged 65 and older at baseline, those in the Q3, Q4 and Q5 dietary choline intake groups had significantly lower risks of cognitive decline by 42.4% (95% CI: 0.381, 0.870), 38.8% (95% CI: 0.394, 0.948) and 39.8% (95% CI: 0.369, 0.985) (*p*-trend = 0.2735), respectively, in the fully adjusted model (Figure 1). The results for dietary PC were similar to those for choline (*p*-trend = 0.8202 for Model 3, Figure 2). The highest quintile of GPC intake was associated with a significant reduced risk of cognitive decline (HR: 0.468, 95% CI: 0.283, 0.771), and a significant trend (*p*-trend = 0.0448) across quintiles of dietary GPC intake was observed in Model 1 (Figure 3). However, the association was marginally significant after adjusting for all covariates (HR: 0.612, 95% CI: 0.344, 1.089, *p*-trend = 0.5758).

### 3.4. Association of Dietary Choline and Its Compounds with Incident Cognitive Decline by Gender

Among males, dietary choline intake in both Q2 and Q3 was associated with a 31.9% (95% CI: 0.489, 0.949) and 34.5% (95% CI: 0.462, 0.929) lower risk of cognitive decline relative to Q1 in Model 3 (*p*-trend = 0.8662, Figure 1). No significant association was found for dietary PC intake (*p*-trend = 0.6954 for Model 3, Figure 2). For dietary GPC, intake in Q2 was associated with a 39.8% (95% CI: 0.425, 0.852) reduction in the risk of cognitive decline compared to Q1 after full adjustment (Figure 3), and a marginally significant association was found in Q4 (HR: 0.711, 95% CI: 0.482, 1.048, *p*-trend = 0.8082).

In females, both dietary choline and PC intakes in the Q2–Q5 groups were associated with a reduced risk of cognitive decline in each model (Figure 1 and Figure 2), but no significant trends across quintiles of choline intake (*p*-trend = 0.1033) or PC (*p*-trend = 0.5491) were observed in Model 3. However, dietary GPC intake did not significantly affect the risk of cognitive decline in females (*p*-trend = 0.1525 for Model 3, Figure 3).

## 4. Discussion

A growing body of evidence suggests that adequate choline intake plays a critical role in neurodevelopment and lifelong brain function during the first 1000 days of life. However, the potential for higher choline intake to influence cognition during childhood, adulthood and age-related cognitive decline remains inconclusive [34,35]. As the global population ages, the number of individuals experiencing cognitive decline is expected to increase, raising concerns about risk factors and potential interventions for age-related neurodegenerative diseases. Several studies have indicated that dietary choline or choline supplementation may improve cognitive function and slow the progression of AD and dementia in older adults [15,16,18], but evidence from Chinese populations is limited. The present study found that higher dietary choline intake (median values: 137.1 mg/d, 177.2 mg/d, 221.5 mg/d and 302.6 mg/d in Q2–Q5 groups, respectively) and PC intake (median values: 118.5 mg/d, 154.3 mg/d and 218.8 mg/d in Q3–Q5, respectively) were associated with a decreased risk of incident cognitive decline in Chinese adults aged 55 and older, particularly among females and adults aged ≥ 65 at baseline. Furthermore, higher GPC intake may help prevent incidence of cognitive decline in adults aged ≥ 65 (median value: 29.3 mg/d in Q5) and males (median value: 20.3 mg/d in Q4). These findings help to address a gap in the literature concerning Chinese populations.

Cognition encompasses a range of higher mental functions, including thinking, reasoning, problem solving, and decision making, which rely on the integration and interaction of more basic processes such as perception, learning, memory, and emotion [36]. These complex cognitive functions are carried out in the brain. Since 1998, choline has been recognized as an essential nutrient with its metabolites playing both structural and regulatory roles in the body [35]. It is well established that choline is acetylated to form acetylcholine, which is a neurotransmitter involved in learning, memory and attention. The loss of cholinergic neurons is linked to cognitive impairment, particularly memory loss and AD. Choline may also influence brain function through its metabolite betaine, which is involved in epigenetic regulation [35,37]. Diet is the primary source of choline, and inadequate intake can lead to deficiency. However, the relationship between dietary choline and cognitive health in humans remain unsolved. 

Several cohort studies, including those using the Framingham Offspring Cohort, have examined the role of dietary choline in cognitive function and the risk of AD and dementia [16,38]. One study found that nondemented individuals aged 36–83 years who had higher concurrent choline intake (mean: 321.1 mg/d) performed better on verbal (β = 0.60, 95% CI: 0.29, 0.91) and visual memory (β = 0.66, 95% CI: 0.19, 1.13). In addition, higher long-term choline intake (mean: 322.7 mg/d) was negatively associated with white-matter hyperintensity (WMH) volume (β = −0.05, 95% CI: −0.10, −0.01), which is a marker of cognitive decline and AD [38]. Further research based on this cohort revealed that low choline intake (≤219 mg/d for dementia and ≤215 mg/d for AD) was associated with a higher risk of developing dementia and AD in participants free of these conditions at baseline [16]. In contrast, studies using cohorts from NHANES (≥60 years) [17] and the Kuopio Ischaemic Heart Disease Risk Factor Disease Study (42–60 years) [15] did not find a protective effect of dietary choline against cognitive decline or dementia. 

Due to limited data on choline content in the Chinese Food Composition database, few studies have explored dietary choline intake and its impact on health in China. Our recent work has focused on understanding choline intake in the Chinese population and investigating its potential neuroprotective effects in middle-aged and older adults. Huang et al. found that higher dietary choline intake improved cognitive performance and delayed cognitive decline in females using longitudinal data from the CHNS on individuals aged 55–79 years [39]. Similarly, Guan et al. found that higher concurrent choline intake was significantly associated with better global cognitive function (β = 0.083, 95% CI: 0.046, 0.119) and lower odds of poor cognition (OR = 0.762, 95% CI: 0.676, 0.860) in adults aged ≥ 55 years from the CHNS cohort (1997–2018) with particularly strong effects in females and individuals aged 55–65 years [40]. Given the lower choline intake levels in the Chinese population [39,40] and the variability in individual dietary habits, our study further demonstrated that cumulative average choline intake, reflecting long-term dietary status, was significantly associated with a decreased risk of cognitive decline in Chinese adults aged ≥ 55 years, particularly among females and individuals aged ≥ 65 years. These findings are largely consistent with previous studies [39,40] and suggest that higher dietary choline intake may benefit age-related cognitive function by improving global cognitive performance and preventing cognitive impairment. 

While the evidence on the relationship between dietary choline intake and cognitive function is still debated, it is clear that the findings vary across population-based studies from different countries [16,17,38,39,40]. These discrepancies may be due to differences in the study sample characteristics, methodologies, ethnicity, and dietary patterns. Intervention studies have demonstrated that choline supplementation can improve several cognitive functions in healthy older adults, including verbal memory, executive function and language fluency [18,41]. Therefore, larger well-designed population studies are needed to confirm the potential of dietary choline in preventing cognitive decline and improving cognitive deficits.

PC, a major dietary source of choline, is thought to have the potential to improve brain function in individuals with dementia and similar cognitive impairments. However, a systematic review found no significant benefit of PC supplementation in patients with AD or other forms of dementia, suggesting a lack of clinical efficacy [42,43]. On the other hand, a notable benefit of PC was observed in individuals with subjective memory disturbances in a study conducted in the 1980s [44]. These findings imply that PC may be effective in the preclinical phase of dementia, and further studies using standardized cognitive assessments are needed to confirm this hypothesis. 

In contrast to PC, GPC has shown modest improvements in cognitive dysfunction associated with neurodegenerative and vascular dementia [43]. GPC has been used as both a medication and nutraceutical to enhance cognitive function in individuals with neurological conditions, including dementia. However, discrepancies remain regarding its approval as a prescription medicine and inconsistencies about its effectiveness across different countries [45]. In 2023, Sagaro et al. addressed the efficacy of GPC in treating cognitive impairment in patients with adult-onset neurological disorders by pooling seven randomized controlled trials and one prospective cohort study. The study found improvements in cognitive function following GPC treatment (1200 mg/d) either alone or in combination with donepezil (1200 mg/d GPC) [45], providing strong evidence regarding the safety of GPC for patients with neurological conditions. However, the impact of GPC on age-related cognitive decline in the general population remains underexplored. 

Our group has previously investigated the longitudinal association between dietary GPC intake and cognitive function, finding a positive correlation between higher GPC intake and better cognitive performance especially among males [40]. This study is the first to examine the potential of dietary PC and GPC in reducing the risk of cognitive decline in the Chinese population. We observed that long-term higher PC intake significantly reduced the risk of incident cognitive decline in the overall population, particularly among female and those aged ≥ 65 years at baseline. This finding is similar to the effects of dietary choline observed in the present study. In contrast, the benefits of higher dietary GPC intake were marginal with a reduced risk observed in males (Q4) and those aged ≥ 65 years (Q5) at baseline. These findings provided important evidence supporting PC as a beneficial nutrient for cognitive performance [46], suggesting that higher PC intake could serve as an intervention for cognitive impairment during aging in Chinese adults. 

The modest effects of GPC observed in this study may be attributable to the relatively low levels of choline and GPC intake in the study population, as GPC intake was much lower than the dose used in intervention studies [45]. Higher intake may be necessary to achieve more pronounced effects. In comparison, the average dietary choline intake for healthy adults ranges 284–468 mg/d for males and 263–374 mg/d for females across different countries with intake in adults aged ≥ 65 years averaging 266–392 mg/d [11]. Dietary PC intake in the US population aged 45–75 years was 166–214 mg/d for males and 132–179 mg/d for females based on data from the Multiethnic Cohort Study (1993–1996). For GPC, the intake was 53–70 mg/d for males and 42–58 mg/d for females [47]. The Framingham Heart Study Offspring Cohort (1991–1995) reported average PC and GPC intakes as 159.6 mg/d and 54.4 mg/d, respectively, in participants (with a mean age of 54.5 years) [16]. In contrast, the average dietary intakes in the present study were significantly lower: 188.0 mg/d for choline, 126.7 mg/d for PC, and 17.1 mg/d for GPC. This suggests that choline intake, particularly GPC intake, is lower in Chinese adults, which can largely be explained by differences in dietary patterns between China and Western countries. 

Other factors may also contribute to the marginal significance of the findings. For instance, a better diet quality has been associated with higher choline intake, as compared to those with lower intake [17]. This could serve as a potential confounder, as other nutrients, such as fiber and omega-3 polyunsaturated fatty acids, might play a stronger neuroprotective role in cognitive function in older adults. Large-scale, population-based longitudinal studies from various countries are needed to further clarify the efficacy and safety of GPC intake and supplementation for cognitive health from middle to late life.

This study has several limitations. First, the CHNS relied on 24 h dietary recalls, which are susceptible to recall and social desirability bias [17]. Additionally, the food composition dataset used for dietary choline assessment was not fully based on Chinese food items, and choline supplementation was not measured, which have introduced some inaccuracies in estimating choline intake. While we accounted for a broad range of covariates in our models, potential confounders such as genetic factors, family history, environmental exposures, and cognitive training may not have been fully controlled for. Furthermore, we did not assess the full range of cognitive domains, which may limit the sensitivity of our findings regarding the effects of dietary choline and its components. Additionally, the lack of data on hearing capability could have influenced cognitive assessments. Finally, the findings of the present study may not be generalizable to other populations due to differences in race, age and dietary habits. The relatively young population (73% aged 55–64 years) and the short follow-up (5.8 years) may limit the ability to assess long-term cognitive trajectories. Future studies should include precise biomarkers of choline intake, assess a broader range of cognitive tasks, involve diverse populations with longer follow-up, and utilize neuroimaging techniques.

## 5. Conclusions

This study highlights age and gender differences in the protective associations between dietary choline, PC and GPC intake and incident cognitive decline in middle-aged and older adults. Specifically, higher choline and PC intake were associated with a reduced risk of cognitive decline in females and older adults, while higher GPC intake showed benefits for males and older adults. However, the overall lower intake of choline and its compounds in the Chinese population may limit the potential benefits of dietary GPC. It is recommended that individuals in mid- and late life increase their consumption of choline-rich foods or dietary supplements, alongside a balanced diet, to maintain cognitive health and delay the onset of cognitive impairment during aging.

## Figures and Tables

**Table 1:** Baseline characteristics in subjects by quintiles of dietary choline.

Parameters	Total (n = 4502)	Total Choline *				
		Q1 (0.1–128.6 mg/d, n = 916)	Q2 (82.6–169.8 mg/d, n = 865)	Q3 (123.3–208.4 mg/d, n = 861)	Q4 (164.0–261.6 mg/d, n = 911)	Q5 (225.2–954.8 mg/d, n = 949)
Age (years)	62.3 ± 6.2	61.9 ± 6.0 ^a^	62.2 ± 6.2 ^a^	62.0 ± 6.2 ^a^	62.1 ± 5.9 ^a^	63.1 ± 6.6 ^b^
Gender						
male	2317 (51.5)	422 (46.1)	431 (49.8)	415 (48.2)	510 (56.0)	539 (56.8)
female	2185 (48.5)	494 (53.9)	434 (50.2)	446 (51.8)	401 (44.0)	410 (43.2)
Educational level						
≤primary school	2210 (49.1)	554 (60.5)	492 (56.9)	438 (50.9)	394 (43.3)	332 (35.0)
middle school	1127 (25.0)	223 (24.3)	201 (23.2)	223 (25.9)	242 (26.6)	238 (25.1)
≥high school	1165 (25.9)	139 (15.2)	172 (19.9)	200 (23.2)	275 (30.2)	379 (39.9)
Household income level ^#^						
low	1382 (30.7)	473 (51.6)	322 (37.2)	245 (28.5)	197 (21.6)	145 (15.3)
medium	1504 (33.4)	288 (31.4)	319 (36.9)	325 (37.8)	319 (35.0)	253 (26.7)
high	1616 (35.9)	155 (16.9)	224 (25.9)	291 (33.8)	395 (43.4)	551 (58.1)
Region						
central	1280 (28.4)	310 (33.8)	286 (33.1)	260 (30.2)	213 (23.4)	211 (22.2)
eastern	2031 (45.1)	289 (31.6)	315 (36.4)	360 (41.8)	472 (51.8)	595 (62.7)
western	1191 (26.5)	317 (34.6)	264 (30.5)	241 (28.0)	226 (24.8)	143 (15.1)
Urbanization level ^#^						
low	1469 (32.6)	544 (59.4)	341 (39.4)	254 (29.5)	203 (22.3)	127 (13.4)
medium	1541 (34.2)	215 (23.5)	332 (38.4)	327 (38.0)	347 (38.1)	320 (33.7)
high	1492 (33.1)	157 (17.1)	192 (22.2)	280 (32.5)	361 (39.6)	502 (52.9)
Current smoking						
yes	1292 (28.7)	266 (29.0)	263 (30.4)	214 (24.9)	285 (31.3)	264 (27.8)
no	3210 (71.3)	650 (71.0)	602 (69.6)	647 (75.2)	626 (68.7)	685 (72.2)
Alcohol intake						
yes	1421 (31.6)	251 (27.4)	272 (31.5)	255 (29.6)	314 (34.5)	329 (34.7)
no	3081 (68.4)	665 (72.6)	593 (68.6)	606 (70.4)	597 (65.5)	620 (65.3)
Physical activity level ^#^						
low	1278 (28.4)	190 (20.7)	207 (23.9)	247 (28.7)	278 (30.5)	356 (37.5)
medium	1569 (34.9)	273 (29.8)	271 (31.3)	309 (35.9)	341 (37.4)	375 (39.5)
high	1655 (36.8)	453 (49.5)	387 (44.7)	305 (35.4)	292 (32.1)	218 (23.0)
CVD history						
yes	1089 (24.2)	174 (19.0)	190 (22.0)	204 (23.7)	252 (27.7)	269 (28.4)
no	3413 (75.8)	742 (81.0)	675 (78.0)	657 (76.3)	659 (72.3)	680 (71.7)
BMI (kg/m^2^)	23.8 ± 3.5	23.2 ± 3.7 ^a^	23.5 ± 3.6 ^a,b^	23.7 ± 3.5 ^b^	24.2 ± 3.4 ^c^	24.2 ± 3.2 ^c^
Dietary intake						
energy (kcal/d)	2228.9 ± 597.6	2037.7 ± 569.8 ^a^	2127.2 ± 550.0 ^b^	2190.1 ± 536.3 ^c^	2305.7 ± 571.9 ^d^	2467.9 ± 650.3 ^e^
choline (mg/d)	188.0 ± 99.7	77.5 ± 28.2 ^a^	130.2 ± 22.2 ^b^	170.5 ± 21.8 ^c^	219.0 ± 22.3 ^d^	333.3 ± 88.7 ^e^
PC (mg/d)	126.7 ± 81.1	42.0 ± 22.7 ^a^	80.8 ± 23.5 ^b^	112.4 ± 24.4 ^c^	148.7 ± 26.0 ^d^	242.2 ± 78.5 ^e^
GPC (mg/d)	17.1 ± 10.8	8.7 ± 4.8 ^a^	12.8 ± 5.2 ^b^	16.2 ± 6.7 ^c^	20.3 ± 8.0 ^d^	26.8 ± 14.6 ^e^
Global cognitive score	17.3 ± 4.7	16.4 ± 4.6 ^a^	17.1 ± 4.5 ^b^	17.0 ± 4.5 ^b^	17.8 ± 4.7 ^c^	18.0 ± 4.9 ^c^

Continuous variables were presented as mean ± SD; otherwise, n (%) was shown. Different superscripted letters mean significant differences in distribution by quintiles of dietary choline intake. * Minimum and maximum of each quintile of cumulative average intake of total choline were shown. ^#^ Mean ± SD in low, medium and high groups were 3431.9 ± 3628.8 yuan, 10,589.0 ± 8669.8 yuan, and 25,065.6 ± 21,306.7 yuan for annual household income per capital, 21.6 ± 20.1 MET·h/w, 95.9 ± 55.9 MET·h/w, and 345.1 ± 182.6 MET·h/w for total physical activity, 46.7 ± 12.1, 71.9 ± 10.3, and 87.8 ± 7.5 for urbanization level, respectively.

## Data Availability

The CHNS datasets used during the current study are partly available at http://www.cpc.unc.edu/projects/china/data after submitting the registration form.
